# Supplementary material for: Early-onset gynecological tumors in DNA repair-deficient xeroderma pigmentosum group C patients: a case series
Source: Commun Med (Lond). 2023 Aug 11;3:109. doi: 10.1038/s43856-023-00341-6 (PMC10421935; doi:10.1038/s43856-023-00341-6)
Supplement: Supplementary file 1 — Description of Additional Supplementary Files [file 43856_2023_341_MOESM1_ESM.pdf]

Description of Additional Supplementary Files

File Name: Supplementary Data 1

Description: The source data to reproduce the figures
