# Supplementary material for: Early-onset gynecological tumors in DNA repair-deficient xeroderma pigmentosum group C patients: a case series
Source: Commun Med (Lond). 2023 Aug 11;3:109. doi: 10.1038/s43856-023-00341-6 (PMC10421935; doi:10.1038/s43856-023-00341-6)
Supplement: Supplementary file 4 — Reporting Summary [file 43856_2023_341_MOESM4_ESM.pdf]

nature portfolio

Corresponding author(s): Sergey Nikolaev

Last updated by author(s): Jul 29, 2023

Reporting Summary

Nature Portfolio wishes to improve the reproducibility of the work that we publish. This form provides structure for consistency and transparency in reporting. For further information on Nature Portfolio policies, see our Editorial Policies and the Editorial Policy Checklist.

Statistics

For all statistical analyses, confirm that the following items are present in the figure legend, table legend, main text, or Methods section.

n/a	Confirmed	
☐	☒	The exact sample size (n) for each experimental group/condition, given as a discrete number and unit of measurement
☐	☒	A statement on whether measurements were taken from distinct samples or whether the same sample was measured repeatedly
☐	☒	The statistical test(s) used AND whether they are one- or two-sided<br>*Only common tests should be described solely by name; describe more complex techniques in the Methods section.*
☒	☐	A description of all covariates tested
☒	☐	A description of any assumptions or corrections, such as tests of normality and adjustment for multiple comparisons
☐	☒	A full description of the statistical parameters including central tendency (e.g. means) or other basic estimates (e.g. regression coefficient) AND variation (e.g. standard deviation) or associated estimates of uncertainty (e.g. confidence intervals)
☒	☐	For null hypothesis testing, the test statistic (e.g. F, t, r) with confidence intervals, effect sizes, degrees of freedom and P value noted<br>*Give P values as exact values whenever suitable.*
☒	☐	For Bayesian analysis, information on the choice of priors and Markov chain Monte Carlo settings
☒	☐	For hierarchical and complex designs, identification of the appropriate level for tests and full reporting of outcomes
☒	☐	Estimates of effect sizes (e.g. Cohen's d, Pearson's r), indicating how they were calculated

Our web collection on statistics for biologists contains articles on many of the points above.

Software and code

Policy information about availability of computer code

Data collection	No software was used for data collection
Data analysis	QC of the reads: FASTQC (Andrews, 2015) (v0.11.7); Read mapping: BWA-MEM (v0.7.12) software (Li and Durbin, 2009); BAM files preparation: samtools (Li et al., 2009) (v1.9); variant calling: GATK mutect2 (Depristo et al., 2011) (v4.0.10.1); annotation of the variants: oncotator (Ramos et al., 2015) (v1.9.9.0), mutational signature analysis: MutationalPatterns software (Blokzijl et al., 2018) (v.1.11.0), SigProfilerMatrixGenerator v.1.0 software (Bergstrom et al., 2019), R statistical software v3.5.1; SCNA analysis: FACETS v0.5.14 (Shen and Seshan, 2016).

For manuscripts utilizing custom algorithms or software that are central to the research but not yet described in published literature, software must be made available to editors and reviewers. We strongly encourage code deposition in a community repository (e.g. GitHub). See the Nature Portfolio guidelines for submitting code & software for further information.

Data

Policy information about availability of data

All manuscripts must include a data availability statement. This statement should provide the following information, where applicable:

- Accession codes, unique identifiers, or web links for publicly available datasets
- A description of any restrictions on data availability
- For clinical datasets or third party data, please ensure that the statement adheres to our policy

The dataset generated during the current study (WGS FASTQ files and corresponding somatic VCF files) is available from the corresponding author on reasonable request after approval by the data access committee due to the data privacy of the patients. The source data to reproduce the figures is available in Supplementary Data 1 file. Genomic datasets of XP-C leukemia and sporadic OGCT tumors used in this study are available in the European Genome-Phenome Archive (EGA) under accession codes EGAS00001004511 and EGAS00001004249 respectively, access is restricted and can be granted under approval by the data access committees.

Research involving human participants, their data, or biological material

Policy information about studies with human participants or human data. See also policy information about sex, gender (identity/presentation), and sexual orientation and race, ethnicity and racism.

Reporting on sex and gender	The sex of all the patients was reported. Gender was not considered and reported in the study.
Reporting on race, ethnicity, or other socially relevant groupings	We reported country of origin for all the patients.
Population characteristics	The study participants were patients with Xeroderma pigmentosum and gynecologycal cancers. This is a very rare hereditary disease and diagnosis of XP and skin cancer were the major indication for the inclusion to the study.
Recruitment	The participants were approached during routine clinical procedures. There was no specific selection of the patients except their diagnosis and consent. We do not anticipate a bias in recruitment of participants in this study
Ethics oversight	This study was approved by the French Agency of Biomedicine (Paris, France), the Ethics Committee from the CPP of the University Hospital of Bordeaux (Bordeaux, France), the Institutional Review Board of Gustave Roussy (CSET: 2018-2820; Gustave Roussy, Villejuif, France). Informed signed consents to publish the case details including identifiable information were obtained from patients and/or their parents according to the Declaration of Helsinki and the French law.

Note that full information on the approval of the study protocol must also be provided in the manuscript.

Field-specific reporting

Please select the one below that is the best fit for your research. If you are not sure, read the appropriate sections before making your selection.

☒ Life sciences ☐ Behavioural & social sciences ☐ Ecological, evolutionary & environmental sciences

For a reference copy of the document with all sections, see nature.com/documents/nr-reporting-summary-flat.pdf

Life sciences study design

All studies must disclose on these points even when the disclosure is negative.

Sample size	We did not predetermine sample size. All the available material was used in the study.
Data exclusions	No data was excluded, all the available samples from relevant cohorts were used.
Replication	We did not attempt to replicate our results based on the sequencing of XP tumors because this is a rare disease and material is not available. Only independent biological replicates were used in this study. No technical replicates were used.
Randomization	The groups were allocated according to the diagnosis (corresponding XP group)
Blinding	The blinding was not applicable to our data. All the researchers acknowledged the grouping of the samples. The blinding was not relevant for this study because we worked with a collection and analysis of rare samples and their comparison with published cohorts.

Reporting for specific materials, systems and methods

We require information from authors about some types of materials, experimental systems and methods used in many studies. Here, indicate whether each material, system or method listed is relevant to your study. If you are not sure if a list item applies to your research, read the appropriate section before selecting a response.

Materials & experimental systems

n/a	Involved in the study	
☒	☐	Antibodies
☒	☐	Eukaryotic cell lines
☒	☐	Palaeontology and archaeology
☒	☐	Animals and other organisms
☐	☒	Clinical data
☒	☐	Dual use research of concern
☒	☐	Plants

Methods

n/a	Involved in the study	
☒	☐	ChIP-seq
☒	☐	Flow cytometry
☒	☐	MRI-based neuroimaging

Clinical data

Policy information about clinical studies

All manuscripts should comply with the ICMJE guidelines for publication of clinical research and a completed CONSORT checklist must be included with all submissions.

Clinical trial registration	This study is not a part of clinical trials and patients did not participate in the clinical trials
Study protocol	The study comprises a retrospective multicentric case series study. The study protocol was not created for this study because it was not registered as clinical trials.
Data collection	The data was collected by the the doctors in the period from 2005-2022
Outcomes	The outcomes of the patients are described in the Results of the manuscript.
